# Characterization of a decellularized pericardium extracellular matrix hydrogel for regenerative medicine: insights on animal-to-animal variability

**DOI:** 10.3389/fbioe.2024.1452965

**Published:** 2024-08-14

**Authors:** Dalila Di Francesco, Elena Marcello, Simona Casarella, Francesco Copes, Pascale Chevallier, Irene Carmagnola, Diego Mantovani, Francesca Boccafoschi

**Affiliations:** ^1^ Laboratory for Biomaterials and Bioengineering, Canada Research Chair Tier I for the Innovation in Surgery, Department of Min-Met-Materials Engineering and Regenerative Medicine, CHU de Quebec Research Center, Laval University, Quebec, QC, Canada; ^2^ Department of Health Sciences, University of Piemonte Orientale “A. Avogadro”, Novara, Italy; ^3^ Department of Mechanical and Aerospace Engineering, Politecnico di Torino, Torino, Italy; ^4^ Polito BioMed Lab, Politecnico di Torino, Torino, Italy

**Keywords:** decellularized extracellular matrix, hydrogel, bovine pericardium, characterization, regenerative medicine, animal-to-animal variability

## Abstract

In the past years, the use of hydrogels derived from decellularized extracellular matrix (dECM) for regenerative medicine purposes has significantly increased. The intrinsic bioactive and immunomodulatory properties indicate these materials as promising candidates for therapeutical applications. However, to date, limitations such as animal-to-animal variability still hinder the clinical translation. Moreover, the choice of tissue source, decellularization and solubilization protocols leads to differences in dECM-derived hydrogels. In this context, detailed characterization of chemical, physical and biological properties of the hydrogels should be performed, with attention to how these properties can be affected by animal-to-animal variability. Herein, we report a detailed characterization of a hydrogel derived from the decellularized extracellular matrix of bovine pericardium (dBP). Protein content, rheological properties, injectability, surface microstructure, *in vitro* stability and cytocompatibility were evaluated, with particular attention to animal-to-animal variability. The gelation process showed to be thermoresponsive and the obtained dBP hydrogels are injectable, porous, stable up to 2 weeks in aqueous media, rapidly degrading in enzymatic environment and cytocompatible, able to maintain cell viability in human mesenchymal stromal cells. Results from proteomic analysis proved that dBP hydrogels are highly rich in composition, preserving bioactive proteoglycans and glycoproteins in addition to structural proteins such as collagen. With respect to the chemical composition, animal-to-animal variability was shown, but the biological properties were not affected, which remained consistent in different batches. Taken together these results show that dBP hydrogels are excellent candidates for regenerative medicine applications.

## 1 Introduction

In the last decade, hydrogels are among the most widely used biomaterials for regenerative medicine applications. They are three dimensional (3D), highly hydrated viscoelastic networks made up of natural and/or synthetic polymers (Singh et al., 2018). Their structure forms through either a physical self-assembly of the components or by chemical crosslinking. This process is referred to as gelation, during which the polymer solution passes from a “sol” to “gel” state. What makes hydrogels such appealing biomaterials for regenerative medicine is the fact that their 3D swollen structure mimics the physiological cellular environment, represented by the extracellular matrix (ECM) (González-Díaz and Varghese, 2016). Moreover, most hydrogels can be injected, thus providing a minimally invasive route of administration, and once they are in gel-state they can optimally fill the space of oddly shaped defects. Finally, hydrogels can be tailored to properly suit their application and can be used as drug delivery systems (Hoffman, 2002; Ahmed, 2015).

Recently, two biomaterials properties have gained importance in the use of hydrogels for regenerative medicine: bioactivity and immunomodulation (Singh and Peppas, 2014; Flégeau et al., 2017; Adu-Berchie and Mooney, 2020). These properties refer to the ability of biomaterials to not simply be inert and evade the immune system, but to interact with the surrounding cellular environment and guide it towards a favourable response for tissue regeneration. In particular, immunomodulation is referred as the ability to modulate the immune system and counteract its over-activations and under-activations. In the case of biomaterials, the immunomodulatory potential should limit an excessive activation of the immune system as a response to the biomaterial implantation, thereby limiting inflammation and enhancing tissue repair and regeneration (Khan and Reddy, 2014; Mokarram and Bellamkonda, 2014). Some biomaterials are intrinsically bioactive and immunomodulatory, while others can be developed to achieve these properties, for example, by adding bioactive molecules or by designing the material in a specific way (Hotaling et al., 2015; Bu et al., 2022). In fact, these properties highly depend on the materials characteristics, both physical, such as ultrastructure and mechanical properties, and chemical, such as the material’s composition. Some hydrogels, particularly from natural origins, have superior intrinsic bioactivity, including immunomodulatory potential, due to their composition. This innate advantage has been proven countless times in hydrogels derived from decellularized extracellular matrix (dECM) (Fishman et al., 2013; Sicari et al., 2014; Huleihel et al., 2017; Keane et al., 2017b; Petrosyan et al., 2017; Hong et al., 2020; Tan et al., 2021).

dECM can be obtained from different tissues and animal sources through the process of decellularization, which seeks to completely remove the cellular and antigenic components, while preserving the native composition and architecture of ECM. To then process dECM into a hydrogel, the material is usually dried, milled into powder, solubilized enzymatically, and gelled using temperature and/or pH-neutralization to reassemble its molecular bonds. dECM hydrogels differ from one another depending on their animal and/or tissue of origin, and the protocols used to produce them. For these reasons, any hydrogel produced from dECM needs to be thoroughly characterized in terms of physical, chemical and biological properties (Saldin et al., 2017; Brown et al., 2022; Kort-Mascort et al., 2023). However, what remains common to all dECM hydrogels are the superior advantages of their use in regenerative medicine: first, the composition is varied and rich, providing a diversity of exploitable biochemical cues; secondly, the solubilization process used to obtain the hydrogel releases and exposes several bioactive components, such as cell signalling peptides motifs and extracellular vesicles bound to the matrix; finally, the 3D structure of the hydrogel mimics the physiological environment, allowing for easy integration in the host, cell infiltration, growth, and remodelling. Due to these reasons, dECM hydrogels have been successfully employed in different regenerative medicine applications (Meng et al., 2015; Tukmachev et al., 2016; Faust et al., 2017; Sawkins et al., 2018; Seo et al., 2018; Su et al., 2018; Bordbar et al., 2020; Chen et al., 2020; Ozudogru et al., 2021; Xu et al., 2021). Regardless, the clinical translation of these products has been greatly hampered by the main disadvantage of dECM materials: animal-to-animal variability. It is well known that many factors can affect the physical and chemical properties of tissue ECM, such as animal age, sex, environmental conditions, disease, etc. This leads to the impossibility to always obtain the same exact tissue of origin for dECM production (Sicari et al., 2012; Hernandez et al., 2020; Saldin et al., 2021). For this reasons, animal-to-animal variability cannot be completely controlled and represents an obstacle for the success of the biomaterial clinical translation and commercialization. In this context, it is important to have an in-depth characterization of each dECM-derived hydrogel, and to understand how animal-to-animal variability may affect the physical, chemical, and biological properties of the hydrogel, thus its performance in regenerative medicine applications (Hernandez et al., 2020).

Among dECM biomaterials, decellularized bovine pericardium (dBP) has been broadly studied and used for regenerative medicine purposes, with commercially available products, especially as surgical membranes (Jara et al., 2015; Mazzitelli et al., 2015; Strange et al., 2015; Olsen et al., 2016; Williams et al., 2021; Eyuboglu et al., 2024). The success of this dECM biomaterials is due to different reasons, such as the fact that dBP is a tissue characterized by low cellularization, thus, easy, optimized, and standardized decellularization are well known. Furthermore, dBP products have shown to be highly compatible for different applications, and that they retain bioactive components, like the recently discovered matrix bound nanovesicles (Umashankar et al., 2013; Stieglmeier et al., 2021; Xing et al., 2021; Di Francesco et al., 2024). Worth noting is also the fact that bovine pericardium is a by-product of the food industry, thus its use as a starting material in tissue engineering and regenerative medicine promotes the concept of circular economy. dBP has studied in different biomaterial forms, particularly as patches and crosslinked scaffolds for cardiovascular applications, however, little is known on the properties of hydrogel form (Oswal et al., 2007; Bielli et al., 2018; Alhadrami et al., 2019). While dBP hydrogels have been shown to have strong regenerative potential (Bracaglia et al., 2017; Carton et al., 2021; Di Francesco et al., 2022), there are limited studies evaluating their physical and chemical properties. Moreover, if animal-to-animal variability affects these properties, and, thus, the performance of the hydrogel, is still unknown. Herein, we present a detailed characterization of the rheological, injectability, microstructure, composition, *in vitro* stability, and biocompatibility properties of dBP hydrogels. Moreover, the possible effects of animal-to-animal variability affecting major chemical, physical and biological characteristics were evaluated.

## 2 Materials and methods

### 2.1 Hydrogel preparation

To represent animal-to-animal variability, three out of nine representative decellularized bovine pericardia (dBP.1, dBP.2, dBP.3) were used for experiments, kindly provided by Tissuegraft Srl (Alessandria, Italy) (Italian patent number 102020000007567; International patent number PCT/IB2021/052779). Tissuegraft Srl was not involved for the analysis of the provided dBP samples. Briefly, the dBPs was lyophilized and milled, then enzymatically digested to obtain hydrolysed solutions (pre-gel). The dBP hydrogel was formed by allowing gelation at 37°C for 30 min. The material is used at the maximum concentration of 9 mg/mL, unless otherwise specified.

### 2.2 Proteomic analysis

The protein concentration of dBP.1, dBP.2, and dBP.3 hydrogels was determined using bicinchoninic acid assay (BCA) (Pierce, Thermo Scientific, IL, United States). 50 μg of proteins were diluted in Laemmli Sample Buffer (62.5 mM Tris-HCl pH 6.8, 10% glycerol, 5% beta-mercaptoethanol, 0.005% bromophenol blue, 2% Sodium Dodecyl Sulphate) (Sigma Aldrich, Milan, Italy) and separated by Sodium Dodecyl Sulphate-PolyAcrylamide Gel Electrophoresis (SDS-PAGE) using a 10% N,N′-methylenebisacrylamide (acrylamide) gel. The electrophoresis was stopped once the samples had run 1 cm of length, and the gel was stained with Coomassie Blue (Sigma Aldrich, Milan, Italy).

Protein digestion and mass spectrometry analyses were performed by the Proteomics Platform of the CHU de Québec Research Center (Quebec, QC, Canada). Briefly, gel bands of interest were cut into small pieces. Proteins were reduced with 10 mM DTT (ditiotritol) and alkylated with 55 mM iodoacetamide. Trypsin digestion was performed using 126 nM of modified porcine trypsin (Sequencing grade, Promega, Madison, WI) at 37°C for 18 h. Digestion products were extracted using 1% formic acid, 2% acetonitrile followed by 1% formic acid, 50% acetonitrile. The recovered extracts were pooled, vacuum centrifuge dried and then resuspended into 15 µL of 2% acetonitrile, 0.05% trifluoric acid and 5 µL were analysed by mass spectrometry.

Samples were analyzed by nano LC-MS/MS using a Dionex UltiMate 3,000 nanoRSLC chromatography system (Thermo Fisher Scientific) connected to an Orbitrap Fusion mass spectrometer (Thermo Fisher Scientific, San Jose, CA, United States). Peptides were trapped at 20 μL/min in loading solvent (2% acetonitrile, 0.05% TFA) on a 5 mm × 300 μm C18 pepmap cartridge pre-column (Thermo Fisher Scientific/Dionex Softron GmbH, Germering, Germany) for 5 min. Then, the pre-column was switched online with a Pepmap Acclaim column (ThermoFisher) 50 cm × 75 µm internal diameter separation column and the peptides were eluted with a linear gradient from 5%–40% solvent B (A: 0.1% formic acid, B: 80% acetonitrile, 0.1% formic acid) in 30 min, at 300 nL/min. Mass spectra were acquired using a data dependent acquisition mode using Thermo XCalibur software version 4.3.73.11. Full scan mass spectra (350–1,800 m/z) were acquired in the orbitrap using an AGC target of 4e5, a maximum injection time of 50 ms and a resolution of 1,20,000. Internal calibration using lock mass on the m/z 445.12003 siloxane ion was used. Each MS scan was followed by MSMS fragmentation of the most intense ions for a total cycle time of 3 s (top speed mode). The selected ions were isolated using the quadrupole analyser in a window of 1.6 m/z and fragmented by Higher energy Collision-induced Dissociation (HCD) with 35% of collision energy. The resulting fragments were detected by the linear ion trap at a rapid scan rate with an AGC target of 1e4 and a maximum injection time of 50 ms. Dynamic exclusion of previously fragmented peptides was set for a period of 20 s and a tolerance of 10 ppm. MGF peak list files were created using Proteome Discoverer 2.3 software (Thermo). MGF sample files were then analysed using Mascot (Matrix Science, London, United Kingdom; version 2.5.1). Mascot was set up to search a contaminant database and Uniprot *Bos taurus* Reference proteome (37510 entries, UP000009136), assuming semi-trypsin as the digestion parameter and with a fragment ion mass tolerance of 0.60 Da and a parent ion tolerance of 10.0 PPM. Scaffold (version Scaffold_5.1.2, Proteome Software Inc., Portland, OR) was used to validate MS/MS based peptide and protein identifications. Peptide identifications were accepted if they could be established at greater than 95% probability by the Scaffold Local FDR algorithm. Protein identifications were accepted if they could be established at greater than 95%. Protein probabilities were assigned by the Protein Prophet algorithm (Nesvizhskii et al., 2003). Proteins that contained similar peptides and could not be differentiated based on MS/MS analysis alone were grouped to satisfy the principles of parsimony. Results as presented as number of exclusive unique peptide count.

### 2.3 Rheological characterization

In order to characterize the gelation kinetics and viscoelastic behaviour of different dBP hydrogel concentrations (dBP.1, dBP.2, and dBP.3), time and frequency sweep tests were performed using a Modulator Compact Rheometer MCR 302 (Anton Paar Anton Paar Italia S.r.l, Italy) with a parallel 25 mm plate and temperature control. For time sweep analysis, the rheometer was set to 4°C, 500 μL of dBP pre-gel were loaded, and distilled water was placed around the plate to avoid sample drying. Parameters were set at 0.5% strain and 1 Hz frequency. The temperature was ramped from 4°C to 37°C in 2 min, and data recorded for 15 min (until and after plateau reaching). For frequency sweep analysis, temperature and strain were kept constant at 37°C and 0.5%, respectively, and angular frequency was changed from 0.1 to 100 rad/s. Mean shear storage modulus (G′) value was taken at 1 rad/s. For viscosity measurement, 500 μL of dBP pre-gel were loaded on the plate, temperature was kept constant at 25°C and shear rate was changed from 0.1 to 1,000 s^−1^. Analysis was carried for 10 min. The analyses were performed in triplicate for each dBP hydrogel concentration (9–4 mg/mL dBP) and each sample batch.

### 2.4 Injectability

In order to evaluate the dBP hydrogel’s injectability, differences in gelation kinetics and shear storage (G′) modulus of the hydrogel were evaluated for extruded and non-extruded dBP hydrogels, using the ElastoSens™ Bio2 (Rheolution instruments, Canada). The ElastoSens™BIO2 technique measures the shear storage and loss moduli of hydrogels as a function of time and/or temperature, in real-time and in a non-destructive manner. The analysis is based on the gentle mechanical vibration of a sample confined in a sample holder and the response is detected by a laser. 7 mL of 9 mg/mL dBP pre-gel from dBP.1, dBP.2, and dBP.3 were loaded in the sample holder and test was immediately performed. For injectability testing, pre-gels were subject to injection forces by extruding them through syringe needles using a syringe pump. 7 mL of dBP pre-gel was loaded into a 10 mL Luer lock syringe and pumped through a 25G, 1/2″ needle (7018345, Nordson EFD, United States). The syringe pump (Cole Parmer Canada Company, Canada) was set at fixed flow rate of 6 mL/min, and the pre-gel was extruded directly into the ElastoSens™ Bio2 sample holder, test was then immediately run. Differences in gelation kinetics and viscoelastic properties of the extruded and non-extruded dBP hydrogels were evaluated by performing the test in soft mode, at 37°C, and the gelation and viscoelastic properties were monitored for 1 h 30 min. Data was provided by the Soft Matter Analytics™ (Rheolution instruments, Canada) and analysed in Excel.

### 2.5 Microstructure characterization

For scanning electron microscopy (SEM) analysis, 9 mg/mL dBP hydrogel samples were fixed overnight at 4°C in 2.5% cold glutaraldehyde. Samples were then washed, dehydrated with an ethanol series (30%–50%–70%–90%–100% ethanol) for 40 min per incubation, then left in ethanol 100% overnight. After a 6 h distilled water wash, samples were freeze dried with an −80°C incubation and overnight lyophilization. Samples were sputter coated twice with gold-palladium (Polarion SC500 Sputter Coater) then imaged with a by a FEI QUANTA 250 (Oregon, United States) SEM equipped with a tungsten filament and operated in the high-vacuum mode with an acceleration voltage of 15 kV. Image analysis was performed with Mountains^®^ software (Digital Surf, Besancon, France) on representative images of three different dBP batch hydrogel samples.

### 2.6 Stability and degradability

In order to assess the *in vitro* stability and degradation kinetics, dBP hydrogels from different batches were prepared in 0.4 μm pore Corning^®^ Transwell^®^ polyester membrane inserts (SigmaAldrich CLS3470, Italy) and allowed to gel at 37°C for 30 min. The empty transwells and transwells with hydrogels were weighted. A solution of 1 mg/mL Collagenase Type I (C0130, Sigma-Aldrich, Italy) in Tris buffer (Tris-HCl 0.5 M, 5 mM CaCl_2_, pH 7.4) was used to perform enzymatic degradation, while phosphate buffered saline (PBS) was used as control aqueous medium. The solutions were place in the bottom side of the 24 multiwell plates, and transwells containing hydrogels were placed in the solutions and incubated at 37°C. For 14 days, the transwells with hydrogels were weighted at different time points. Solutions were renewed every 48 h.

### 2.7 Cell culture

Immortalized human mesenchymal stromal cells (hMSCs) (clonal cell line Y201 (James et al., 2015)) were used for cell viability assay. Cells were cultured in 75 cm^2^ flasks, maintained in Dulbecco’s Modified Eagle Medium Low Glucose (DMEM) (ECM0749L, Euroclone, Milan, Italy), supplemented with 5 mM glutamine (Sigma-Aldrich 1294808, Milan, Italy), 1% penicillin, streptomycin and amphotericin-B (PSF) (Euroclone, Milan, Italy), and 15% foetal bovine serum (FBS) (Gibco, Milan, Italy).

### 2.8 Cell viability

The cytocompatibility of dBP.1, dBP.2, and dBP.3 hydrogels towards hMSCs was evaluated using MTS assay (Promega Italia Srl, Milan, Italy). First, 200 μL of three different dBP batches were placed in 48-well plates and allowed to gel by leaving at 37°C for 30 min hMSCs were seeded on the dBP hydrogels at a concentration of 5,000 cells/well. Cells seeded directly in the well served as controls. At time points of 1 and 7 days after cell seeding, MTS assay was performed by discarding culture media and adding 250 μL MTS solution, composed of 4:5 DMEM without phenol red (Cytiva SH30585.02, Fisher scientific, Milan, Italy) and 1:5 MTS solution, according to manufacturer’s protocol. The 490 nm absorbance was read using Victor 4X Multilabel Plate Reader (Perkin Elmer, Milan, Italy) and data were analysed in Excel (Microsoft, Redmond, WA, United States). Experiments were performed in triplicate.

### 2.9 Statistical analysis

Sample average and standard deviation were calculated on experimental triplicates, then, a one tail Student’s T statistical test for homoscedastic samples was performed on the software InStat3 (Graphpad Instat Software Inc., United States) to verify the statistical significance (significance level of 5%). The differences between variables with a value of *p* < 0.05 were considered statistically significant.

## 3 Results

### 3.1 Proteomic analysis

**FIGURE 1:**
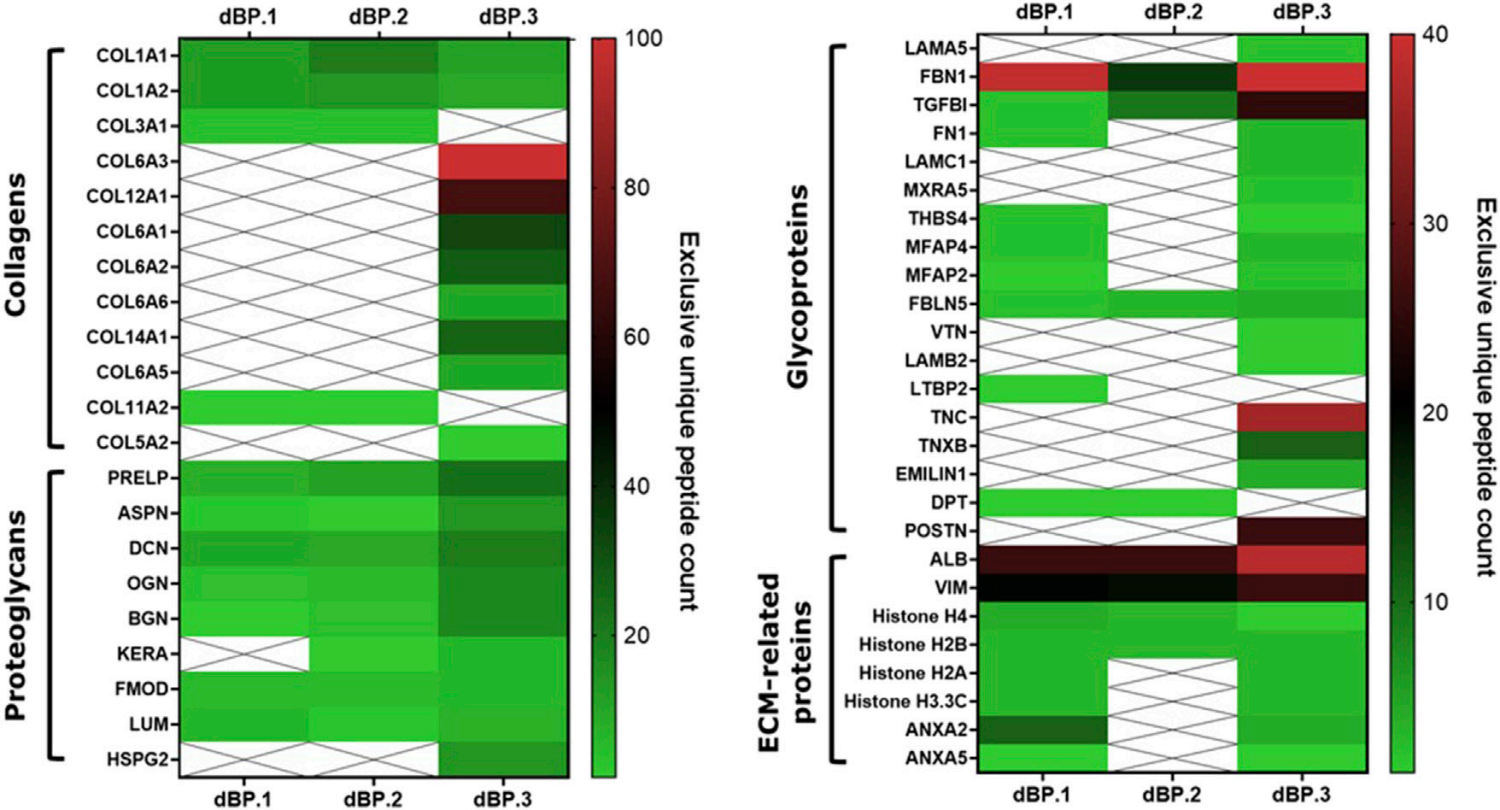
Heatmap of matrisome peptides found in three different batches of dBP hydrogels.

Proteomic analysis was performed on dBP.1, dBP.2, and dBP.3 hydrogels and results indicate that the hydrogels retain a rich and complex composition, preserving native components of the ECM (Figure 1). A total of 47 different core matrisome and ECM-related peptides were found, 17 of which were common across the three batches. Among these were major structural core matrisome peptides, such as type I collagen chains (COL1A1 and COL1A2), proteoglycans, like prolargin (PRELP), decorin (DCN), asporin (ASPN), biglycan (BGN), fibromodulin (FMOD), lumican (LUM) and mimecan (OGN), matrix glycoproteins, like microfibril’s fibrillin-1 (FBN1), fibulin-5 (FBLN5) and transforming growth factor, beta-induced (TGFBI), and finally other ECM related peptides, such as histones. While two batches (dBP.1 and dBP.2) showed very similar protein composition, dBP.3 seemed to be the only one retaining a higher amount of elements in both qualitative and quantitative terms. For instance, dBP.3 also expressed core matrisome peptides such as type VI collagen (COL6), laminins (LAMA5, LAMC1 and LAMB2), tenascins (TNC and TNXB) and periostin (POSTN). Only collagen type XI collagen (COL11A2) and dermatopontin (DPT) were retained in dBP.1 and dBP.2 but not in dBP.3. Moreover, dBP.1 also preserved glycoproteins found in dBP.3, such fibronectin 1 (FN1), microfibril associated proteins (MFAP) and thrombospondin 4 (THBS4), while these were lost in dBP.2, which showed to be the batch lacking most peptides retained in the other batches. Finally, Latent-transforming growth factor beta-binding protein 2 (LTBP2) was the only peptide found singularly in dBP.1.

### 3.2 Rheological characterization

**FIGURE 2:**
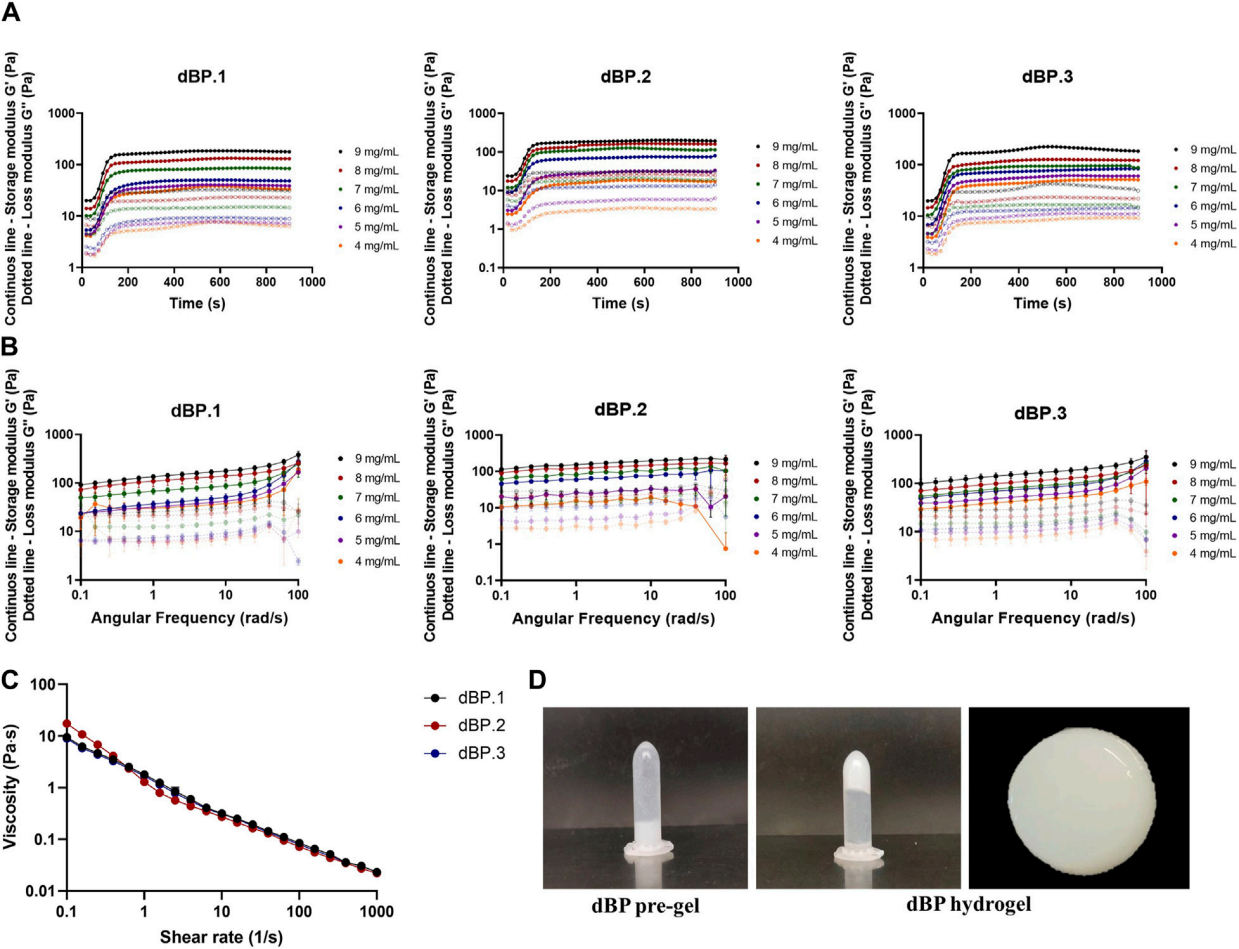
Rheological properties of dBP hydrogels. **(A)** Gelation kinetics. Time-sweep curves of batches dBP.1, dBP.2, and dBP.3 at different hydrogels concentrations. Data are presented as average of experimental triplicate mean. **(B)** Frequency sweep curves of batches dBP.1, dBP.2, and dBP.3 at different hydrogels concentrations. Data are presented as average of experimental triplicate mean and SD. **(C)** Viscosity of three different batches of 9 mg/mL dBP hydrogels. **(D)** Tube inversion of 9 mg/mL dBP gelation before (pre-gel) and after gelation (hydrogel), with macroscopic image of dBP hydrogel.

Hydrogel concentrations from 4 to 9 mg/mL, obtained from dBP.1, dBP.2, and dBP.3, were characterized in order to evaluate gelation kinetics, viscoelastic properties and injectability. Gelation kinetics were evaluated through time sweep analysis, increasing the temperature from 4°C to 37°C. For all the concentrations evaluated (Figure 2A), a solid-*like* state (*G′* > *G″*) was detected at the begging of the analysis, in agreement with previous studies. Both G′ and G″ showed a rapid increase, reaching a plateau within 5 min (Sawkins et al., 2013; Giobbe et al., 2019; Fernandez-Carro et al., 2024). Results were visually confirmed by tube inversion test, showing a thermally formed hydrogel able to preserve its shape (Figure 2D). The three different batches of dBP showed similar gelation kinetics, especially for the highest concentrations investigated (i.e., 7, 8 and 9 mg/mL) showing juxtaposable gelation curves. Frequency sweep analyses were performed on all dBP hydrogel concentrations investigated, as shown in Figure 2B, in which separate data of dBP.1, dBP.2, and dBP.3 batches are displayed. All dBP hydrogel concentrations showed ability to form a hydrogel with gel-like behaviour, since G′ was notably higher than G″. Both moduli showed a slight dependence on frequency, especially for high frequencies values (above 50 rad/s). This behavior was more pronounced for samples with a low concentration (4, 5 and 6 mg/mL), suggesting the formation of weaker hydrogels. All the three batches exhibited an increase in G′ with increase in hydrogel concentration, ranging from 29 ± 13 Pa for 4 mg/mL hydrogels to 145 ± 20 Pa for 9 mg/mL hydrogels. Slight variations between dBP.1, dBP.2, and dBP.3 were detected for low polymer concentrations (4, 5 and 6 mg/mL), while at high concentrations (i.e., 7, 8, and 9 mg/mL) the G′ values of the different batches were comparable.

**FIGURE 3:**
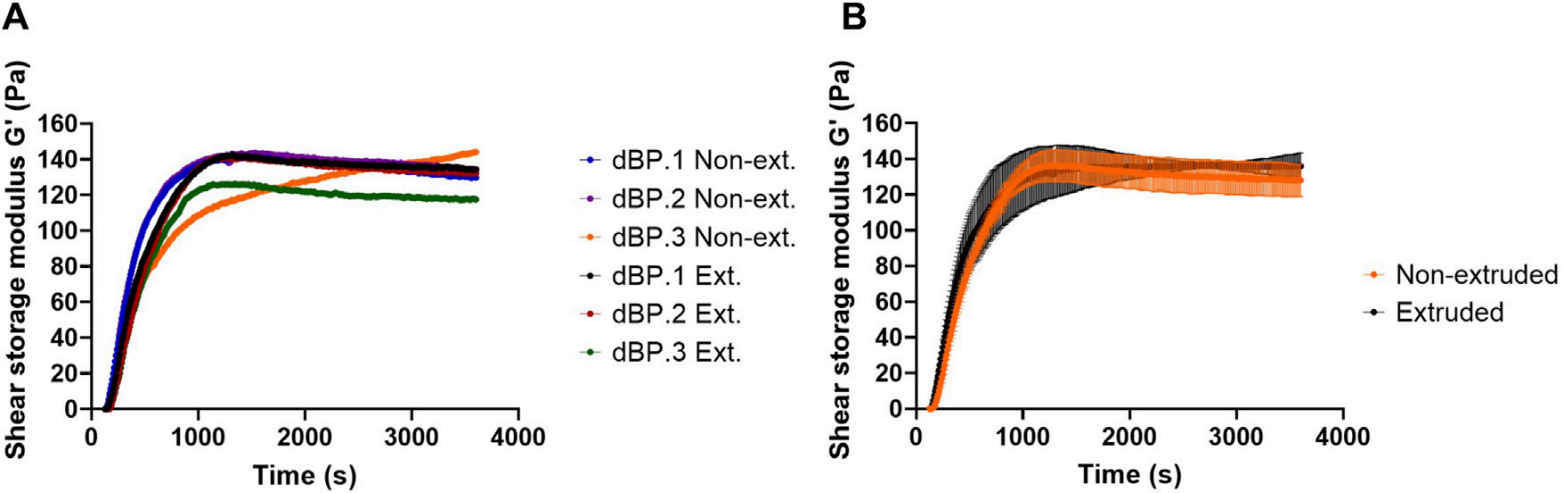
Injectability evaluation of 9 mg/mL dBP hydrogel. **(A)** Gelation kinetics of non-extruded (Non-ext.) and (Ext.) extruded 9 mg/mL dBP hydrogels derived from dBP.1, dBP.2, and dBP.3 are shown singularly. **(B)** Gelation kinetics of non-extruded and extruded dBP hydrogels presented as average data from three different dBP batches, with SD.

Due to the fact that 9 mg/mL is the highest achievable dBP concentration, and rheological results show that this is able to produce a stable and soft hydrogel, this concentration was chosen for the further investigation of dBP hydrogels. Hydrogel viscosity of 9 mg/mL pre-gel solution was also assessed, and dBP.1, dBP.2, and dBP.3 batches all presented shear thinning behaviour (Figure 2C). Moreover, the ability of the hydrogel to withstand injection forces, and reassemble without alterations in rheological properties, was analysed using the ElastoSens™ Bio2 method. Figure 3A shows that the gelation kinetics of 9 mg/mL dBP non extruded and extruded hydrogels are similar for dBP.1 and dBP.2. Merged data from the three batches shows juxtaposable gelation curves for the batches (Figure 3B), either extruded or not. The mean G′ at *plateau* showed values of 130 ± 6 Pa for non-extruded and 137 ± 4 Pa for extruded hydrogels, and did not show any significant statistical differences, proving that the hydrogels’ rheological properties were not altered by injection forces.

### 3.3 Microstructure characterization

**FIGURE 4:**
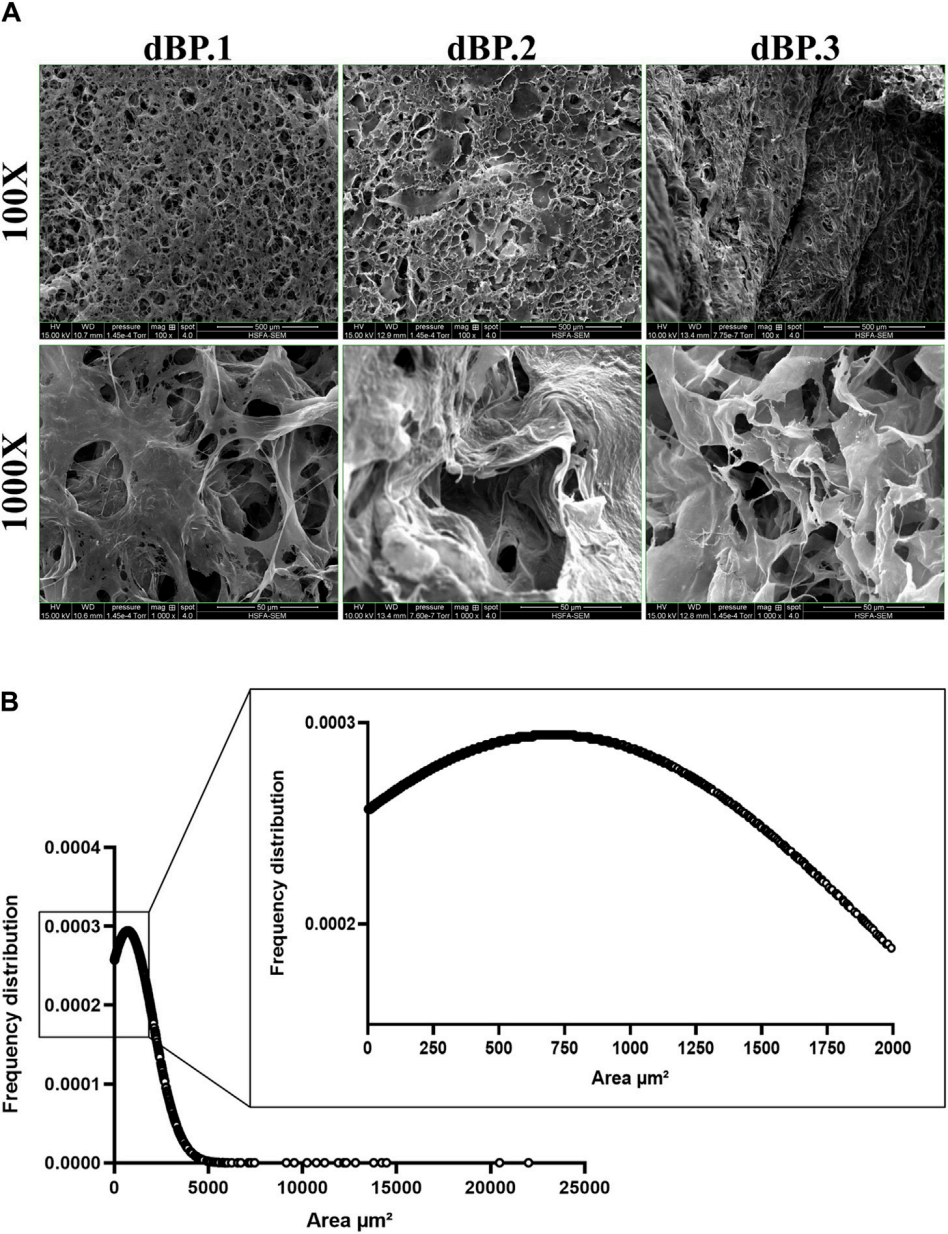
Morphological analysis of 9 mg/mL dBP hydrogels with SEM. **(A)** SEM images of different dBP hydrogel batches at 9 mg/mL. × 100 magnification, scale bar: 500 μm and × 1,000 magnification, scale bar 50 μm are shown. **(B)** Frequency distribution of 9 mg/mL dBP hydrogel pore size.

The surface ultrastructure of 9 mg/mL dBP hydrogels derived from different batches was evaluated using SEM and images were analysed for porosity and pore size of the surfaces. Figure 4 shows that the material’s surface presents a fibrous, interconnective and rough-textured surface. Furthermore, the dBP hydrogel surface shows a structure with 51% (± 5%) of the area covered by pores, as presented in Figure 4A. Pore size analysis shows randomly distributed pore size, ranging from 1.2 to 22,006 μm^2^, and a mean pore size of 709 μm^2^. The frequency distribution of pore size is presented in Figure 4B.

### 3.4 Stability and degradability

**FIGURE 5:**
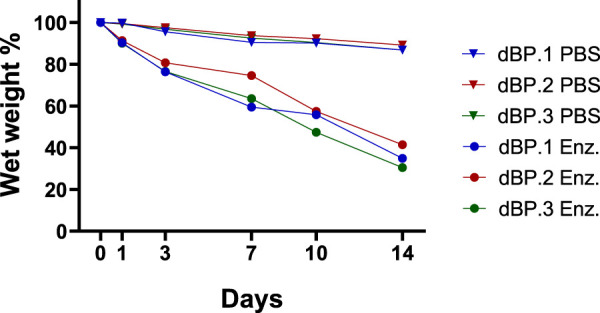
Percentage wet weight variation over time of 9 mg/mL different dBP hydrogels batches (dBP.1, dBP.2, and dBP.3) placed in enzymatic medium (Enz.) or PBS.

The determination of stability and degradation kinetics of 9 mg/mL dBP hydrogels from the different batches was performed both in PBS and enzymatic collagenase type I medium. Results (Figure 5) show that the all the hydrogels are stable up to 2 weeks in PBS medium, while in enzymatic medium a significant amount of weight is lost after 3 days, compared to PBS. By 1 week the hydrogels in enzymatic medium have lost a significant amount of weight compared to time 0, and finally at 2 weeks more than 50% of their weight is degraded. The same stability and degradation behaviour is observed amongst the three dBP hydrogel batches.

### 3.5 Cell viability

**FIGURE 6:**
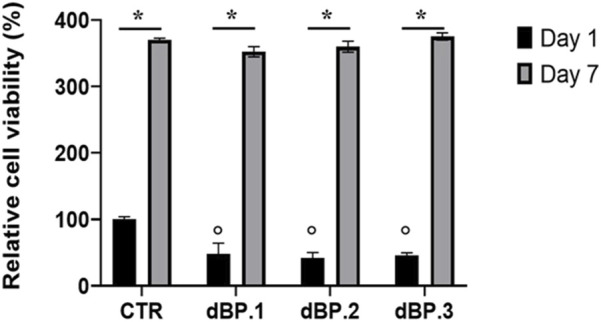
dBP cytocompatibility. MTS assay performed on hMSCs cultured on three different batches of 9 mg/mL dBP hydrogels at 1 and 7 days. *: Day 1 vs. Day 7, °: dBP vs. CTR, *p* < 0.05.

The cytocompatibility of dBP.1, dBP.2, and dBP.3 hydrogel batches towards hMSCs was evaluated. Results (Figure 6) show that dBP hydrogels have significantly lower cell viability at day 1 compared to the control, however, the hydrogels show an increase of relative cell viability by 7 days, comparable to the control. Moreover, dBP hydrogels show to induce a consistent statistically significant increase of relative cell viability from day 1 to day 7 throughout the different dBP batches, showing cytocompatibility.

## 4 Discussion

Hydrogels derived from dECM have recently proven to be excellent candidates for many regenerative medicine applications, thanks to their intrinsic bioactivity and immunomodulatory potential. However, the lack of in-depth characterization of their properties, and how these are affected by animal-to-animal variability, have hampered their translation into clinical practice. Any biomaterial product is subject to variability, caused for example, by material processing protocols or operator handling, and this is particularly true in the research laboratory setting. However, when thinking about large-scale production of biomaterials, either for clinical use and/or commercialization, a tight standardization of manufacturing protocols is required. This minimizes product variability to negligible levels; nonetheless, this process is hindered when considering materials derived from animal tissues, such as dECM (Hernandez et al., 2020; Wang et al., 2022). This is due to the fact that dECM is subject to variability deriving from the animals’ age, sex, species, disease, diet and environmental conditions, etc. It is well known that these factors can greatly affect the tissue properties (Sicari et al., 2012; Sizeland et al., 2014; Dziki et al., 2017b; Capella-Monsonís et al., 2023). Therefore, the general guideline for dECM biomaterials production is to minimize animal variability wherever possible: use the same supplier of animal products, animals of same age, species, sex and grown/nurtured the same way. Still, some variability remains due to uncontrollable factors, making it impossible to obtain the exact same tissue in terms of chemical and physical properties. However, the question remains if these differences are translated into the final biomaterial product, and how these would affect its performance in regenerative medicine applications.

Herein, hydrogels were derived from three out of nine representative different bovine decellularized pericardia to evaluate animal-to-animal variability. Starting bovine pericardium material was obtained from same supplier, ensuring animal regulation and control over age, sex and upbringing environment. Standardized and well-known decellularization and sterile hydrogel production protocols were used (Italian patent number 102020000007567; International patent number PCT/IB2021/052779). Therefore, the possible variability given by material production was considered “negligible,” and only intrinsic animal variability was considered for the purposes of this research.

Hydrogels derived from dECM have been known to possess superior biological performance, compared to other synthetic and natural materials, such as collagen, due to the heterogeneity and complexity of their composition, closely mimicking native ECM (Crapo et al., 2011; Spang and Christman, 2018). Some studies have characterized the proteomic composition of dECM derived hydrogels, all suggesting a tissue specific composition, affected by the decellularization and scaffold preparation methods (Johnson et al., 2016; Xu and Mau, 2019; Yazdanpanah et al., 2021; Kort-Mascort et al., 2023). A previous study reported the high collagen type I content of this dBP hydrogel, and that the decellularization method used in this research allows to preserve other ECM components such as elastin and glycosamminoglycans (GAGs) (Di Francesco et al., 2022). However, there is very limited literature on the composition of dBP hydrogels. The findings reported here show that the dBP hydrogels retain a rich composition, including core matrisome collagens, proteoglycans, glycoproteins and other ECM-related peptides. These components underline the large quantity of biochemical cues that dBP hydrogel would be able to provide to cells, influencing cellular behaviour in terms of proliferation, migration and differentiation (Hynes and Naba, 2012). The proteomic results confirmed that the main structural proteins were retained consistently in dBP.1, dBP.2, and dBP.3, among these collagen type I and a high quantity of proteoglycans containing GAGs were found. Remarkably, small leucine rich proteoglycans, such as decorin, biglycan, mimecan, fibromodulin and lumican were retained. These proteoglycans do not simply play a structural role but have also been recently discovered as regulators of the immune system and angiogenesis (Maiti et al., 2023), and have been used as therapeutic agents for different regenerative medicine applications, such as corneal wound healing (Hill et al., 2018), tendon healing (Pechanec et al., 2020; Leahy et al., 2023; Xu et al., 2023), and skin wound healing (Liu X.-J. et al., 2013; Zheng et al., 2014). Glycoproteins were also preserved in the dBP hydrogels, like fibronectin and TGFBI, which have been also exploited as materials for their potential in regenerative medicine (Parisi et al., 2020; Ruiz et al., 2020). Monteiro-Lobato et al. evaluated the protein composition of ECM derived from differentially digested bovine pericardium. Their results also confirm the very tissue-specific composition of ECM materials, and that digestion methods and timings can affect the final products composition, independently of the origin tissue. Interestingly, their work shows a superior richness and diversity in composition of pericardium derived dECM compared to other tissues, which proves a great biological advantage, many of which peptides were also found in the proteomic analysis reported herein (Monteiro-Lobato et al., 2022). A study by Seif-Naraghi et al. reported the proteomic analysis of dECM derived from pericardium of human and porcine tissue (Seif-Naraghi et al., 2010). They also reported collagen type I to be the main component, as proved for most dECM materials (Saldin et al., 2017; Kort-Mascort et al., 2023). On the other end, the retention of other ECM components was shown to be species-specific. Both human and porcine dECM showed to retain higher quantities of structural collagens but not a big variety of proteoglycans and glycoproteins compared to the results obtained in this study on bovine pericardia hydrogels.

Due to the fact that generally the composition of physiological ECM is complex, dynamic and highly variable, it was expected that this would reflect in animal-to-animal variability on the chemical composition of the dBP hydrogels (Hernandez et al., 2020). In fact, differences in composition between dBP.1, dBP.2, and dBP.3 were noted. One of the batches, dBP.3, retained more ECM content than the other two, and in higher quantities. dBP.3 also showed to retain collagen type V and VI, perlecan, laminins, and tenascins. These peptides are usually found highly associated to muscle tissue and/or as components of the basement membrane of tissues, such as in serous pericardium (Bashey et al., 1992; Chiquet-Ehrismann and Tucker, 2011; Cescon et al., 2015; Kaur and Reinhardt, 2015; Gubbiotti et al., 2017; Shklover et al., 2019; Castells-Sala et al., 2022). The differential expression of such basement membrane components also suggests that tissue harvesting from the product supplier may play a role in the products’ variability. Other peptides were also differentially expressed in the batches. For example, dermatopontin (DPT), a matrisome protein involved in the pathophysiology of cardiac tissue, was expressed in dBP.1 and dBP.2, but not in dBP.3 (Liu X. et al., 2013). Instead, the structural proteoglycan latent transforming growth factor β binding protein 2 (LTBP-2), which plays an elastic role in the pericardium, was only expressed in dBP.2 (Shipley et al., 2000). What is striking, is that these differences in protein composition did not affect the physical and biological properties of the dBP hydrogels. In fact, all dBP hydrogel batches, independently of animal-to-animal variability, showed the same gelation kinetics, injectability, rheological properties, surface structure, and ability to sustain and induce the same increase in cell viability.

dBP hydrogels showed to be thermoresponsive, liquid at room temperature, but able to form a hydrogel in less than 30 min at 37°C, at different concentrations ranging from 4 to 9 mg/mL of dBP. This self-assembly thermoresponsive gelation is maintained even after extrusion of the hydrogel, making the dBP injectable as a pre-gel solution, thus providing a minimally invasive route of administration. This property is also confirmed by the shear thinning behaviour presented by the dBP hydrogel, which is common for dECM hydrogels (Pati et al., 2014; Saldin et al., 2017). The hydrogel concentration is a factor affecting the final G′ and viscoelastic properties, which are shown to increase with the increase of concentration. This behaviour has been already observed for hydrogels derived from dECM and this feature has shown to allow to tailor the hydrogels rheological properties according to the intended application (Freytes et al., 2008; Massensini et al., 2015; Keane et al., 2017a). For high concentration of dBP hydrogels, storage and loss moduli showed little dependence on frequency, while for samples with lower concentrations a higher variability was detected, confirming that concentration hydrogel is a key determinant of the rheological properties. On the other end, even at high frequency, G′ values remained higher than G″ values for all concentrations, further validating the stability of formed hydrogels. At the highest hydrogel concentration (9 mg/mL), the maximum G′ obtained is 145 ± 20 Pa, therefore making dBP hydrogels soft materials, and possible good candidates for soft tissues regenerative medicine applications (Sasikumar et al., 2019). In fact, given the low G′ values achieved with lower dBP hydrogel concentrations, the 9 mg/mL dBP concentration was chosen to carry out further investigations on the characterization, representing also the maximum concentration of dBP that can be provided for biological potential for this particular hydrogel. Noteworthy, the softness of these types of hydrogels has also been shown to promote the immune system towards a wound healing phenotypical response (Bu et al., 2022). Overall, the rheological properties reported here were withing the range of dECM derived hydrogels, usually spanning from a few Pa to 500 Pa, depending on the tissue of origin and dECM content (Saldin et al., 2017). Most importantly, these properties were consistent throughout the different dBP hydrogel batches deriving from different animals, proving that these characteristics are not affected by animal-to-animal variability.

The hydrogels surface of different dBP batches was found to be porous, with a large range pore size, mostly tending to big pores. dECM hydrogels come with different architectures, which can go from dense fibrous gels to porous ones (Wolf et al., 2012; Kao et al., 2021; Yazdanpanah et al., 2021). Moreover, the surface architecture of fresh, non-decellularized bovine pericardium was investigated by Alhadrami et al., which have shown the fibrous nature of this tissue; instead, the hydrogel form reported herein shows how the surface is instead porous once the pericardium is processed into a hydrogel (Alhadrami et al., 2019). The fibrous type of architecture hampers the suitability for cell infiltration and survival, while macroporous hydrogels have shown better suitability for regenerative medicine applications (Lutzweiler et al., 2020). This is due to the fact that porosity and pore size are biomaterial properties that regulate many processes, such as the immune response to biomaterials. Studies by Yin et al. and Yang et al., showed that both inert synthetic and natural scaffolds were able to regulate macrophage polarization and angiogenesis in a pore-size mediated fashion. In both studies, large pores (360–400 µm) led to the induction of a positive regenerative response (Yin et al., 2020; Yang et al., 2023).

The *in vitro* stability and degradability of the dBP hydrogels from different batches were also evaluated. The results established that dBP hydrogels were stable up to 2 weeks in a PBS medium, while the hydrogels started degrading in 7 days when placed in a more physiological-like enzymatic environment. Although the needs of degradation rate are highly dependent on the applications, these type of hydrogels are renowned for their fast degradation rates (Kort-Mascort et al., 2023). However, this quick degradation does not necessarily reflect a cessation of biochemical cues from the biomaterials. Recent discoveries have demonstrated how the degradation of dECM hydrogels leads to the solubilization and release of other strongly bioactive components, further enhancing and prolonging their regenerative potential. In fact, enzymatic degradation and solubilization of dECM expose components such as matrikines and matrix bound nanovesicles, which have potent regenerative and immunomodulatory potential (Maquart et al., 2004; Huleihel et al., 2016; Sivaraman and Shanthi, 2018; Piening and Wachs, 2022; Di Francesco et al., 2024). For these reasons, the regenerative potential of dBP hydrogels biodegradation needs to be further explored, to truly assess the timing length during which these materials provide biochemical stimuli. In the context of clinical translation and industrialization of the product, stability and shelf-life are also critical factors to evaluate. While different dECM material forms, such as powders, are already commercialized and present adequate stability rates, little is known about preserving stable dECM hydrogels as such (Wolf et al., 2012; Saldin et al., 2017; Kort-Mascort et al., 2023). Herein, stability rates of up to 2 weeks in aqueous medium are reported, however, due to the well-known rapid degradation rates of these products, longer stability evaluations should be performed to understand the possibility of achieving an off-the-shelf product for clinical translation.

Finally, as expected all three batches of dBP showed a great cytocompatibility. The strong biocompatibility and regenerative potential of this dBP hydrogel was already reported by our research group, where the immunomodulatory potential of the hydrogel was also shown, coinciding with the many literature reports detailing the biological potential of these hydrogels (Dziki et al., 2017a; Carton et al., 2021; Tan et al., 2021; Di Francesco et al., 2022; Kort-Mascort et al., 2023). In this work, the dBP hydrogels showed to be able not to simply sustain cell viability of hMSCs, but to be able to induce the same statistically significant increase from 1 to 7 days, comparing to that obtained with the control. Importantly, this work provides proof that the variation in chemical composition does not affect the cytocompatibility of the material. A study by Seif-Naraghi et al. evaluated to differences in pericardium hydrogels derived from different human patients, showing that, while basic structural ECM proteins were consistent, the chemical composition varied among the patients. Similarly to what is reported in the present work, the study also demonstrated how this variability was not reflected in the other properties of the hydrogels (Seif-Naraghi et al., 2011). Altogether, these results provide meaningful insights on the characterization and properties of dBP hydrogels, and on how these are affected by animal-to-animal variability. The numerosity of samples for this study was limited to dBP derived from 3 different pericardia, compared to other works evaluating variability, such as those of Seif-Naraghi et al., Sicari et al., and Johnson et al., (Seif-Naraghi et al., 2011; Sicari et al., 2012; Johnson et al., 2016). However, the authors hypothesize that, given the obtained results, while increasing sample number would be pertinent and lead to higher variability in chemical composition, but physical and biological properties remain unaffected; in fact, the dBP hydrogel has been extensively used for regenerative medicine research, showing in all cases extraordinary regenerative potential and a very high reproducibility in terms of biological performances (Carton et al., 2021; Di Francesco et al., 2022). Furthermore, additional information on how the batch differences in chemical composition could impact other cellular processes (i.e., cell differentiation towards a specific lineage) still needs to be properly evaluated. Finally, while in this work dBP hydrogels are provided as sterile for research purposes, the clinical translation of the product requires higher sterile standard, also maintained over time, as indicated in Good Manufacturing Practice and certification protocols, and this still represent a challenge (Golebiowska et al., 2024). In this context, terminal sterilization has been widely researched for these types of hydrogels. This is mainly due to the fact that many different steps occur between animal tissue harvesting and hydrogel production, making it difficult to keep a sterile environment throughout the whole process, thus, it is easier to perform an end-process sterilization step. Among suitable sterilization methods, irradiation, peracetic acid, ethylene oxide and supercritical carbon dioxide are commonly used and have proven successful to achieve good grade sterility. However, research has shown how these methods can importantly impact the hydrogel’s performance in different ways, from changing rheological and mechanical properties, to inducing the loss of different ECM components (Matuska and McFetridge, 2015; White et al., 2018; Tao et al., 2021; Yaldiz et al., 2021; Golebiowska et al., 2024). Thus, the aspect of sterilization methods should be carefully considered and further evaluated for the clinical translation of dECM hydrogels.

The present work provides additional evidence strongly supporting further use of dBP hydrogels in regenerative medicine applications, with a significant increase of the use in clinics in the next future.

## 5 Conclusion

The characterization of hydrogels deriving from different bovine pericardia decellularized extracellular matrix was performed. The dBP hydrogels proved to be extremely rich in protein composition, providing a great variety of biochemical cues. The composition was partially affected by animal-to-animal variability; however, physical and biological properties were absolutely comparable between batches. The obtained hydrogels showed to be porous, injectable, soft, biodegradable, and cytocompatible. Since these properties were consistent throughout different animal source batches, consideration of the potential negligibility of this limitation, that has for a long time hampered their clinical translation, should be considered.

## Figures and Tables

**Table:** 

**ECM**	Extracellular matrix
**dECM**	Decellularized extracellular matrix
**dBP**	Decellularized bovine pericardium
**SEM**	Scanning electron microscopy
**BCA**	Bicinchoninic acid assay
**SDS-PAGE**	Sodium dodecyl sulphate-polyacrylamide gel electrophoresis
**PBS**	Phosphate buffered saline
**hMSCs**	Human mesenchymal stromal cells
**DMEM**	Dulbecco’s modified eagle medium low glucose
**FBS**	Foetal bovine serum
**COL1A1**	Collagen, type I, alpha 1
**COL1A2**	Collagen, type I, alpha 2
**COL3A1**	Collagen, type III, alpha 1
**COL6A3**	Collagen, type VI, alpha 3
**COL12A1**	Collagen, type XII, alpha 1
**COL6A1**	Collagen, type VI, alpha 1
**COL6A2**	Collagen, type VI, alpha 2
**COL6A6**	Collagen, type VI, alpha 6
**COL14A1**	Collagen, type XIV, alpha 1
**COL6A5**	Collagen, type VI, alpha 5
**COL11A2**	Collagen, type XI, alpha 2
**COL5A2**	Collagen, type V, alpha 2
**PRELP**	Prolargin
**ASPN**	Asporin
**DCN**	Decorin
**OGN**	Osteoglycin/Mimecan
**BGN**	Biglycan
**KERA**	Keratocan
**FMOD**	Fibromodulin
**LUM**	Lumican
**HSPG2**	Heparan sulfate proteoglycan 2/Perlecan
**LAMA5**	Laminin, alpha 5
**FBN1**	Fibrillin-1
**TGFBI**	Transforming growth factor, beta-induced
**FN1**	Fibronectin 1
**LAMC1**	Laminin subunit gamma-1
**MXRA5**	Matrix remodeling associated 5
**THBS4**	Thrombospondin 4
**MFAP4**	Microfibril associated protein 4
**MFAP2**	Microfibril associated protein 2
**FBLN5**	Fibulin 5
**VTN**	Vitronectin
**LAMB2**	Laminin, beta 2
**LTBP2**	Latent-transforming growth factor beta-binding protein 2
**TNC**	Tenascin C
**TNXB**	Tenascin XB
**EMILIN1**	Elastin microfibril interfacer 1
**DPT**	Dermatopontin
**POSTN**	Periostin/Osteoblast-specific factor 2
**ALB**	Albumin
**VIM**	Vimentin
**ANXA2**	Annexin A2
**ANXA5**	Annexin A5

## Data Availability

The original contributions presented in the study are included in the article/supplementary material, further inquiries can be directed to the corresponding author.
